# Idiopathic megacolon complicated by life-threatening giant megacolon and respiratory failure due to diaphragmatic eventration: A case report

**DOI:** 10.1016/j.ijscr.2022.107372

**Published:** 2022-06-30

**Authors:** Masataka Fujiwara, Ai Sadatomo, Hiroyoshi Tsubochi, Hisanaga Horie, Alan Kawarai Lefor, Naohiro Sata

**Affiliations:** a Department of Surgery, Division of Gastroenterological, General and Transplant Surgery, Jichi Medical University, 3311 Yakushiji, Shimotsuke, Tochigi 329-0498, Japan; b Division of General Thoracic Surgery, Department of Surgery, Jichi Medical University, 3311 Yakushiji, Shimotsuke, Tochigi 329-0498, Japan

**Keywords:** Idiopathic megacolon, Diaphragmatic eventration, Surgical repair

## Abstract

**Introduction and importance:**

Giant megacolon requiring emergency surgery is rare. Eventration of the diaphragm associated with giant megacolon is also uncommon.

**Case presentation:**

We report a 66-year-old male who presented with abdominal distention and progressive dyspnea. After resuscitation following cardiopulmonary arrest, the patient underwent emergent subtotal abdominal colectomy. Eventration of the diaphragm was found postoperatively and his respiratory condition was insufficient to allow liberation. Plication of both diaphragms was performed through left and right thoracotomy via the 8th intercostal space. Postoperatively the patient made a full recovery.

**Clinical discussions:**

Chronic constipation is a common health condition. A life-threatening condition secondary to chronic constipation is a rarely documented complication. Diaphragmatic eventration that was caused due to chronic megacolon in symptomatic patients requires surgical treatment.

**Conclusions:**

We describe a patient with giant megacolon and diaphragmatic eventration secondary to idiopathic megacolon. The patient underwent subtotal colectomy and diaphragmatic plication and recovered fully.

## Highlights

• A life-threatening condition secondary to chronic megacolon is extremely rare.
• Abdominal compartment syndrome due to giant megacolon resolved by subtotal abdominal colectomy.
• Chronic megacolon from childhood induced eventration of the diaphragm.
• Plication of both diaphragms recovered fully the patient from respiratory dysfunction.

## 1 Background

Idiopathic megacolon is characterized by a permanently enlarged colon in the absence of an identifiable cause. “Giant megacolon” (GMC) is an exacerbated condition of chronic megacolon with a clinical picture of colorectal obstruction with constipation [1]. In line with the Consensus-based Surgical Case Reporting Guideline (SCARE) criteria, we describe a patient with GMC that caused abdominal compartment syndrome requiring emergency surgery and resulted in respiratory failure secondary to eventration of the diaphragm [2].

## 2 Case presentation

A 66-year-old man was transported by ambulance with complaints of progressive abdominal distention and worsening respiratory status. He had abdominal distension and constipation since birth. His symptoms sometimes worsened and were then improved by enema administration. From about age 63, the abdominal distension gradually worsened, and recently he reported difficulty to leave home. Two months before this event, he was seen for evaluation of megacolon. The presence of a rectoanal inhibitory reflex (RAIR) ruled out Hirschsprung's disease and he had no underlying diseases such as obstruction or acute colitis. The RAIR is an anal reflex response characterized by a transient relaxation of the anal canal following distention of the rectum. The major clinical application of RAIR is to diagnose patients with Hirschsprung's disease. In Hirschsprung's disease, the internal anorectal sphincter fails to relax in response to rectal distention [3]. He was finally diagnosed with idiopathic megacolon. Regular medications included polyethylene glycol laxative, which was effective for the treatment of constipation.

**Fig. 1:**
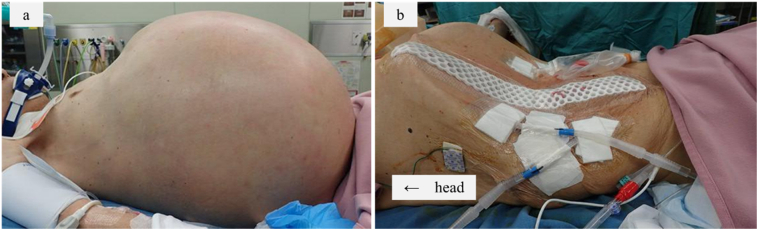
Physical findings. a Abdominal distension before emergency surgery. b The thorax and abdomen were depressed after subtotal colectomy.

**Fig. 2:**
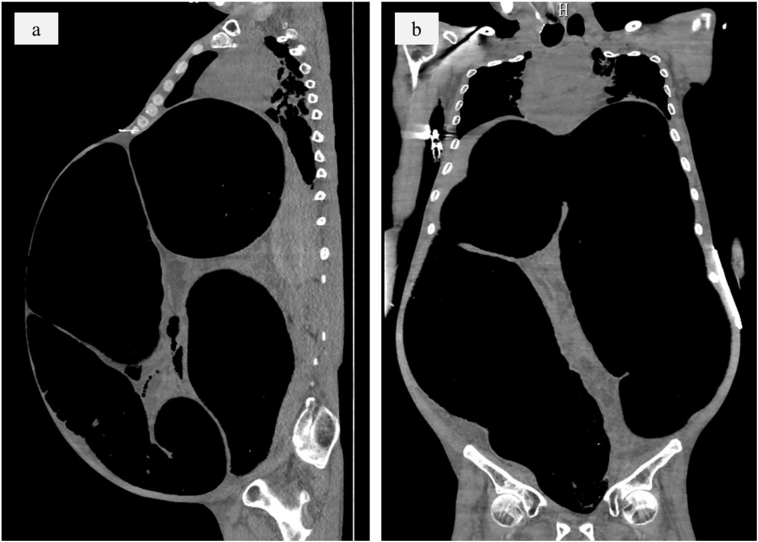
Chest and abdominal computed tomography scan findings. a Sagittal view of the dilated colon filled with gas and feces. b Coronal view revealed an elevated diaphragm which compressed both lungs.

**Fig. 3:**
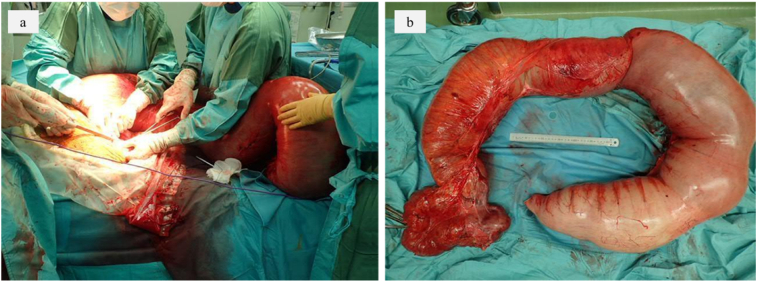
Operative findings. a Dilated colon resection was performed. b Subtotal colectomy specimen removed at laparotomy.

Physical findings at presentation showed he was tachypneic at 109 breaths/min. His oxygen saturation was 73 % on room air and he was intubated immediately. His abdomen was grossly distended to 1.5 times normal size (Fig. 1a). He was in the sitting position during ambulance transfer. When placed in the recumbent position, he became drowsy, and suffered cardiopulmonary arrest. Cardiopulmonary resuscitation was immediately started, and a spontaneous pulse resumed 2 min later. Computed tomography (CT) scan showed a large intestine dilated with gas and feces (Fig. 2a, b). Biochemical investigations revealed worsening renal function and that the patient was acidotic (Table 1). Massive abdominal dilation compromised venous return, and elevated intra-abdominal pressure was suspected. Decompressive laparotomy for abdominal compartment syndrome was required. At surgery, emergent subtotal colectomy and ileostomy creation were performed (Fig. 3a, b). During the operation, a severely dilated ascending to the sigmoid colon and serous ascites were encountered. Perforation and necrosis of colon were not detected. After the operation, the thorax was fixed in the raised position and didn't go down (Fig. 1b). He was brought to the intensive care unit and hemodynamic status improved due to reduced intraabdominal pressure, but pulmonary function was insufficient to allow liberation. Laboratory tests revealed improvement of renal function and acidemia (Table 1). CT scan revealed total eventration of both diaphragms. He couldn't complete spontaneous breathing trial before extubation. The cause of diaphragmatic eventration was suspected to be chronic megacolon since childhood. We decided to perform surgical repair of diaphragmatic eventration caused poor respiratory dysfunction. On the fifth day after operation, bilateral diaphragm plication through left and right thoracotomy via the 8th intercostal space and tracheostomy were performed. After this operation, CT scan revealed improvement of diaphragm movement and good lung expansion. His respiratory condition improved. He was given respiratory rehabilitation for 14 days and liberation from the ventilator was tolerated. He recovered steadily and was transferred to another hospital for further rehabilitation.

**Table. 1:** Blood result.

	On admission day	Day 5		On admission day	Day5
White cell count	13.9 × 10^3^/μl	4.9 × 10^3^/μl	Urea	44 mg/dl	16 mg/dl
Neutrophils	12.0 × 10^3^/μl	3.7 × 10^3^/μl	Creatinine	1.76 mg/dl	0.54 mg/dl
Hemoglobin	14.2 g/dl	7.7 g/dl			
Platelets	317 × 10^3^/μl	127 × 10^3^/μl			
Alanine transaminase	18 U/L	23 U/L	pH	7.124	7.457
Aspartate transaminase	38 U/L	21 U/L	BE	−8.3	8.5
Creatine kinase	812 U/L	236 U/L	Lactate	4.6 mmol/l	0.8 mmol/l
Bilirubin	1.84 mg/dl	0.39 mg/dl			
Albumin	4.8 g/dl	1.8 g/dl

**Fig. 4:**
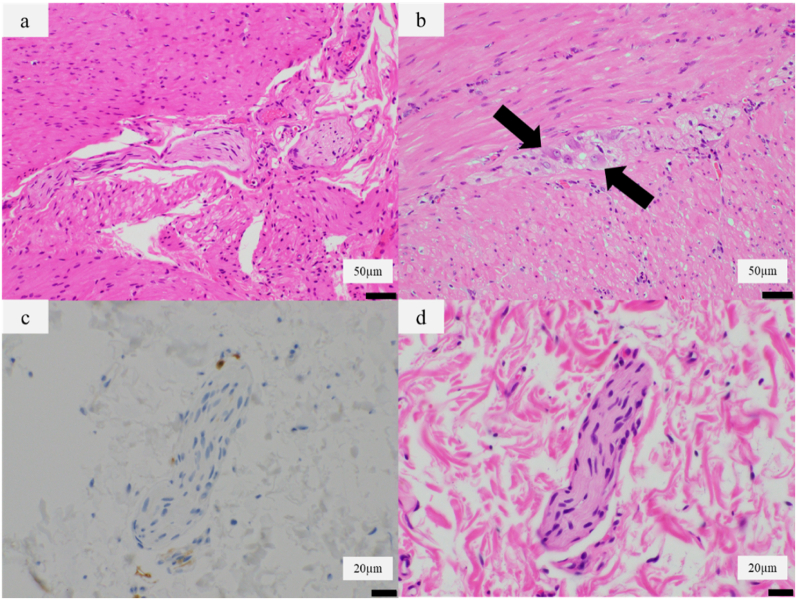
Histopathological findings. a, b Auerbach's and Meissner's plexus showed marked reduction in the number of ganglion cells at the narrow segment of sigmoid colon (a). Some ganglion cells were detected in a normal segment of colon (b). (Hematoxylin–eosin staining, ×20) c, d Sigmoid colon showing negative immunohistochemical staining for Calretinin. (Calretinin immunohistochemistry, ×40), d (Hematoxylin–eosin staining, ×40).

Pathological examination of the resected sigmoid colon showed an absence of ganglion cells (Fig. 4a). Some ganglion cells were observed in the myenteric (Auerbach) and submucosal (Meissner) plexus from the cecum to the descending colon (Fig. 4b). Calretinin immunostaining negative nerve fibers of the sigmoid colon wall were detected (Fig. 4c, d). This pathological examination suggested Hirschsprung's disease, which was contradictory to the result of the rectoanal inhibitory reflex. We concluded that the cause of GMC was an idiopathic disorder related to Hirschsprung's disease.

## 3 Discussion

Megacolon is defined as dilation of the colon caused by a condition other than a mechanical obstruction [4]. The causes of megacolon can be classified into congenital or acquired and the clinical course of megacolon is acute or chronic. Idiopathic megacolon is a rare condition without a detectable cause [5]. The diagnosis of idiopathic megacolon needs exclusion of congenital absence of the nerve plexus (Hirschsprung's disease) and acquired megacolon secondary to an underling systemic disorder. Megacolon in old age patients usually associated with a wide spectrum of illness. Neuropathies (such as Alzheimer's and Parkinson's disease), myocardial infarction, severe infections, electrolyte imbalance/metabolic alterations, surgery or trauma affected megacolon. Chronic megacolon is characterized by symptoms of constipation, abdominal pain, and abdominal distension. Pathologically, it is accompanied by changes in connective tissue, smooth muscle cells, and the enteric nervous system [6], [7], [8], [9]. For 50–70 % of patients with chronic megacolon, non-surgical treatment using laxatives can be effective. Some patients require surgery, such as stoma creation, colectomy or anastomosis and a pull-through procedure because of failed medical therapy [8], [10]. Emergency surgery is required if there is evidence of toxic megacolon, volvulus, or perforation [10], [11].

Eventration of the diaphragm is a condition in which the diaphragm becomes thin and dysfunctional. The causes of eventration of the diaphragm can be classified into congenital or acquired. Congenital eventration of the diaphragm is due to fetal dysgenesis. Acquired eventration of the diaphragm can be caused by phrenic nerve damage, respiratory infection, or exclusion by megacolon and tumors [12], [13], [14]. Nava et al. [14] reported that colectomy improved diaphragmatic function caused by megacolon. The indication for surgical repair of diaphragmatic eventration is symptomatic relief. In the present patient, resection of the megacolon was not sufficient to improve respiratory function. Plication of diaphragm had prevented paradoxical movement and achieved good lung expansion.

In this patient, worsening chronic megacolon caused abdominal compartment syndrome and resulted in cardiopulmonary arrest. To reduce intra-abdominal pressure, emergency surgery was performed. Subtotal colectomy and ileostomy creation were effective to allow stabilization of circulatory status, but respiratory dysfunction due to diaphragmatic eventration became evident. He had no previous history of trauma, surgery, or neoplastic infiltration to phrenic nerve. Diaphragmatic eventration was a result of chronic megacolon since childhood. Bilateral diaphragm plication and tracheostomy were very effective to allow liberation from the ventilator.

## 4 Conclusion

A patient developed abdominal compartment syndrome due to worsening chronic megacolon, also known as GMC, and underwent emergency surgery due to hemodynamic instability. This patient developed diaphragmatic eventration associated with chronic megacolon since childhood and developed improved respiratory function with surgical intervention.

## Sources of funding

This study had no sponsors.

## Ethical approval

This case report about one patient is exempt from ethnical approval.

## Consent

Written informed consent was obtained from the patient for publication of this case report and accompanying images. A copy of the written consent is available for review by the Editor-in-Chief of this journal on request.

## Author contributions

MF, AS and HT performed the operation, and MF, AS, HH and NS managed perioperative care. MF, AS and AL drafted the manuscript. All authors read and approved the final manuscript.

## Registration of research studies

This is not the ‘First in Man’ study.

## Guarantor

Ai Sadatomo is that individual who accepts full responsibility for the work and/or the conduct of the study, had access to the date, and controlled the decision to publish.

## Provenance and peer review

Not commissioned, externally peer-reviewed.

## Declaration of competing interest

The authors declare that there are no competing interests regarding the publication of this paper.

## Appendix A Supplementary data

Supplementary material Image 1
